# 2-Amino-5-methyl­pyridinium 2-amino­benzoate

**DOI:** 10.1107/S1600536812043243

**Published:** 2012-10-24

**Authors:** Kaliyaperumal Thanigaimani, Abbas Farhadikoutenaei, Nuridayanti Che Khalib, Suhana Arshad, Ibrahim Abdul Razak

**Affiliations:** a School of Physics, Universiti Sains Malaysia, 11800 USM, Penang, Malaysia

## Abstract

In the 2-amino­benzoate anion of the title salt, C_6_H_9_N_2_^+^·C_7_H_6_NO_2_^−^, an intra­molecular N—H⋯O hydrogen bond is observed. The dihedral angle between the ring and the CO_2_ group is 8.41 (13)°. In the crystal, the protonated N atom and the 2-amino group of the cation are hydrogen bonded to the carboxyl­ate O atoms *via* a pair of N—H⋯O hydrogen bonds, forming an *R*_2_^2^(8) ring motif. The ion pairs are further connected *via* N—H⋯O hydrogen bonds, resulting in a donor–donor–acceptor–acceptor (*DDAA*) array of quadruple hydrogen bonds. The crystal structure also features a weak N—H⋯O hydrogen bond and a C—H⋯π inter­action, resulting in a three-dimensional network.

## Related literature

For background to the chemistry of substituted pyridines, see: Pozharski *et al.* (1997 ▶); Katritzky *et al.* (1996 ▶). For details of hydrogen bonding, see: Jeffrey (1997 ▶); Scheiner (1997 ▶). For related structures, see: Nahringbauer & Kvick (1977 ▶); Hemamalini & Fun (2010*a* ▶,*b* ▶); Bis & Zaworotko (2005 ▶); Thanigaimani *et al.* (2012 ▶). For hydrogen-bond motifs, see: Bernstein *et al.* (1995 ▶). For hydrogen-bonding patterns in organic salts, see: Baskar Raj *et al.* (2003 ▶). For bond-length data, see: Allen *et al.* (1987 ▶). For stability of the temperature controller used for the data collection, see: Cosier & Glazer (1986 ▶).
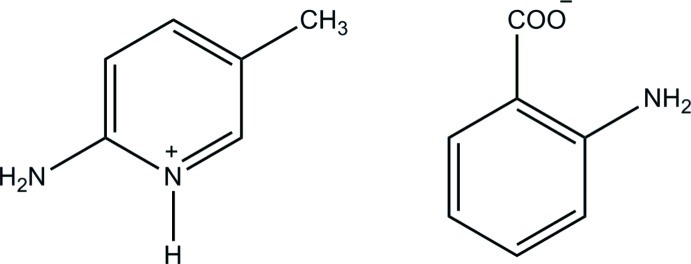


## Experimental

### 

#### Crystal data

C_6_H_9_N_2_^+^·C_7_H_6_NO_2_^−^
*M* *_r_* = 245.28
Monoclinic, 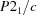

*a* = 9.2394 (8) Å
*b* = 13.9200 (11) Å
*c* = 12.1514 (8) Å
β = 129.850 (4)°
*V* = 1199.82 (16) Å^3^
*Z* = 4
Mo *K*α radiation
μ = 0.09 mm^−1^
*T* = 100 K
0.35 × 0.33 × 0.14 mm

#### Data collection

Bruker SMART APEXII DUO CCD area-detector diffractometer
Absorption correction: multi-scan (*SADABS*; Bruker, 2009 ▶) *T*_min_ = 0.968, *T*_max_ = 0.987
11650 measured reflections
2707 independent reflections
2361 reflections with *I* > 2σ(*I*)
*R*_int_ = 0.031

#### Refinement

*R*[*F* ^2^ > 2σ(*F* ^2^)] = 0.037
*wR*(*F* ^2^) = 0.101
*S* = 1.07
2707 reflections
184 parameters
H atoms treated by a mixture of independent and constrained refinement
Δρ_max_ = 0.27 e Å^−3^
Δρ_min_ = −0.24 e Å^−3^

### 

Data collection: *APEX2* (Bruker, 2009 ▶); cell refinement: *SAINT* (Bruker, 2009 ▶); data reduction: *SAINT*; program(s) used to solve structure: *SHELXTL* (Sheldrick, 2008 ▶); program(s) used to refine structure: *SHELXTL*; molecular graphics: *SHELXTL*; software used to prepare material for publication: *SHELXTL* and *PLATON* (Spek, 2009 ▶).

## Supplementary Material

Crystal structure: contains datablock(s) global, I. DOI: 10.1107/S1600536812043243/is5202sup1.cif

Structure factors: contains datablock(s) I. DOI: 10.1107/S1600536812043243/is5202Isup2.hkl

Supplementary material file. DOI: 10.1107/S1600536812043243/is5202Isup3.cml

Additional supplementary materials:  crystallographic information; 3D view; checkCIF report

## Figures and Tables

**Table 1:** Hydrogen-bond geometry (Å, °) *Cg*1 is the centroid of the C7–C12 ring.

*D*—H⋯*A*	*D*—H	H⋯*A*	*D*⋯*A*	*D*—H⋯*A*
N3—H3⋯O2	0.92 (2)	1.97 (2)	2.6734 (18)	131.8 (15)
N2—H1⋯O1^i^	0.926 (18)	1.982 (18)	2.8561 (14)	157 (2)
N3—H2⋯O1^ii^	0.897 (17)	2.159 (18)	3.0445 (14)	168.7 (14)
N1—H4⋯O2^iii^	0.959 (18)	1.723 (18)	2.6776 (13)	172.7 (17)
N2—H5⋯O1^iii^	0.933 (17)	1.899 (18)	2.8305 (14)	176.8 (16)
C1—H1*A*⋯*Cg*1	0.95	2.58	3.5094 (13)	165

Symmetry codes: (i) 
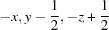
; (ii) 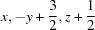; (iii) 
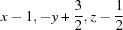
.

**Fig. 1.**
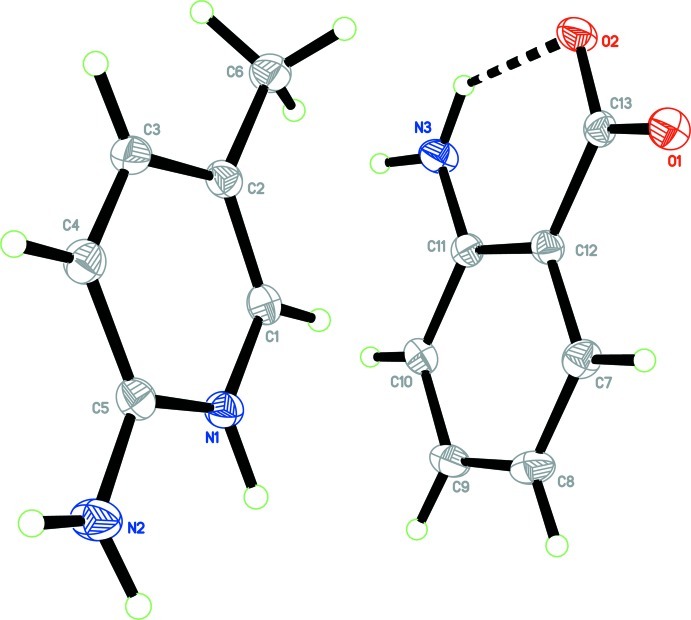
The molecular structure of the title compound with atom labels with 50% probability displacement ellipsoids.

**Fig. 2.**
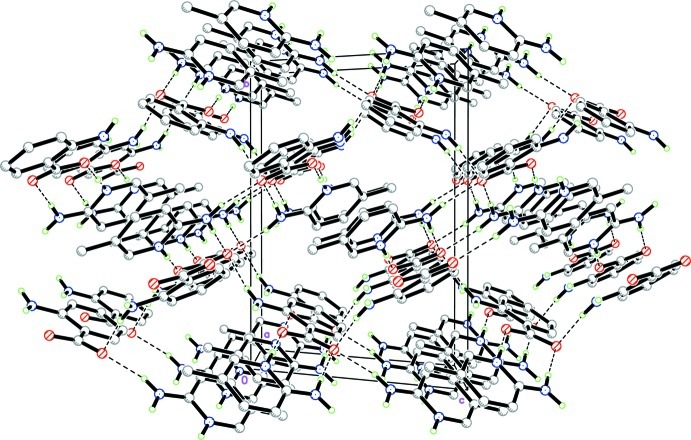
The crystal packing of the title compound. The H atoms not involved in the intermolecular interactions (dashed lines) have been omitted for clarity.

## supplementary crystallographic information

### Comment

Pyridine and its derivatives play an important role in heterocyclic chemistry (Pozharski *et al.*, 1997; Katritzky *et al.*, 1996). The are often involved in hydrogen-bond interactions (Jeffrey, 1997; Scheiner, 1997). The crystal structures of 2-amino-5-methylpyridine (Nahringbauer & Kvick, 1977), 2-amino-5-methylpyridinium 4-hydroxybenzoate (Hemamalini & Fun, 2010*a*), 2-amino-5-methylpyridinium 3-aminobenzoate (Hemamalini & Fun, 2010*b*) and 2-amino-5-methylpyridinium benzoate (Bis & Zaworotko, 2005) have been reported. In order to study some interesting hydrogen bonding interactions, the synthesis and structure of the title compound, (I), is presented here.

The asymmetric unit (Fig. 1) contains one 2-amino-5-methylpyridinium cation and one 2-aminobenzoate anion. In the 2-amino-5-methylpyridinium cation, a wider than normal angle [C1—N1—C5 = 122.86 (13)°] is subtended at the protonated N1 atom. The 2-amino-5-methylpyridinium cation is essentially planar, with a maximum deviation of 0.001 (1) Å for atom C2. The bond lengths (Allen *et al.*, 1987) and angles are normal. In the crystal packing (Fig. 2), the protonated N1 atom and the 2-amino group (N2) are hydrogen-bonded to the carboxylate oxygen atoms (O1 and O2) *via* a pair of intermolecular N1—H4···O2^iii^ and N2—H5···O1^iii^ hydrogen bonds (symmetry code in Table 1), forming an *R*_2_^2^(8) (Bernstein *et al.*, 1995) ring motif. These motifs are centrosymmetrically paired *via* N2—H1···O1^i^ hydrogen bonds (symmetry code in Table 1), forming a complementary donor-donor-acceptor-acceptor (DDAA) array (Baskar Raj *et al.*, 2003). These arrays are further connected *via* N3—H2···O1^ii^ hydrogen bonds (symmetry code in Table 1), resulting a three-dimensional network. There is a typical intramolecular N3—H3···O2 hydrogen bond in the 2-aminobenzoate anion, (graph-set notation S6). The crystal structure is further stabilized by a weak C—H···π interaction (Table 1) involving the C7–C12 (centroid *Cg*1) ring.

### Experimental

Hot methanol solutions (20 ml) of 2-amino-5-methylpyridine (54 mg, Aldrich) and 2-aminobenzoic acid (34 mg, Merck) were mixed and warmed over a heating magnetic stirrer hotplate for a few minutes. The resulting solution was allowed to cool slowly at room temperature and crystals of the title compound (I) appeared after a few days.

### Refinement

N-bound H Atoms were located in a difference Fourier maps and refined freely [refined N—H distances 0.959 (18), 0.926 (18), 0.933 (17), 0.897 (17) and 0.923 (18) Å]. The remaining hydrogen atoms were positioned geometrically (C—H = 0.95–0.98 Å) and were refined using a riding model, with *U*_iso_(H) = 1.2*U*_eq_(C) or 1.5*U*_eq_(methyl C). A rotating group model was used for the methyl group. In the final refinement, eleven outliers were omitted (-4 5 5, -1 4 5, 0 6 6, 4 6 3, 1 6 5, 0 1 1, 3 6 4, 1 4 4, -4 6 7, -1 6 6 and -3 6 7).

### Crystal data

**Table:**

C_6_H_9_N_2_^+^·C_7_H_6_NO_2_^−^	*F*(000) = 520
*M**_r_* = 245.28	*D*_x_ = 1.358 Mg m^−^^3^
Monoclinic, *P*2_1_/*c*	Mo *K*α radiation, λ = 0.71073 Å
Hall symbol: -P 2ybc	Cell parameters from 4117 reflections
*a* = 9.2394 (8) Å	θ = 2.6–32.5°
*b* = 13.9200 (11) Å	µ = 0.09 mm^−^^1^
*c* = 12.1514 (8) Å	*T* = 100 K
β = 129.850 (4)°	Block, pink
*V* = 1199.82 (16) Å^3^	0.35 × 0.33 × 0.14 mm
*Z* = 4	

### Data collection

**Table:**

Bruker SMART APEXII DUO CCD area-detector diffractometer	2707 independent reflections
Radiation source: fine-focus sealed tube	2361 reflections with *I* > 2σ(*I*)
Graphite monochromator	*R*_int_ = 0.031
φ and ω scans	θ_max_ = 27.5°, θ_min_ = 2.9°
Absorption correction: multi-scan (*SADABS*; Bruker, 2009)	*h* = −12→12
*T*_min_ = 0.968, *T*_max_ = 0.987	*k* = −18→18
11650 measured reflections	*l* = −15→15

### Refinement

**Table:**

Refinement on *F*^2^	Primary atom site location: structure-invariant direct methods
Least-squares matrix: full	Secondary atom site location: difference Fourier map
*R*[*F*^2^ > 2σ(*F*^2^)] = 0.037	Hydrogen site location: inferred from neighbouring sites
*wR*(*F*^2^) = 0.101	H atoms treated by a mixture of independent and constrained refinement
*S* = 1.07	*w* = 1/[σ^2^(*F*_o_^2^) + (0.0457*P*)^2^ + 0.4478*P*] where *P* = (*F*_o_^2^ + 2*F*_c_^2^)/3
2707 reflections	(Δ/σ)_max_ < 0.001
184 parameters	Δρ_max_ = 0.27 e Å^−^^3^
0 restraints	Δρ_min_ = −0.24 e Å^−^^3^

### Special details

**Table:**

Experimental. The crystal was placed in the cold stream of an Oxford Cryosystems Cobra open-flow nitrogen cryostat (Cosier & Glazer, 1986) operating at 100.0 (1) K.
Geometry. All e.s.d.'s (except the e.s.d. in the dihedral angle between two l.s. planes) are estimated using the full covariance matrix. The cell e.s.d.'s are taken into account individually in the estimation of e.s.d.'s in distances, angles and torsion angles; correlations between e.s.d.'s in cell parameters are only used when they are defined by crystal symmetry. An approximate (isotropic) treatment of cell e.s.d.'s is used for estimating e.s.d.'s involving l.s. planes.
Refinement. Refinement of *F*^2^ against ALL reflections. The weighted *R*-factor *wR* and goodness of fit *S* are based on *F*^2^, conventional *R*-factors *R* are based on *F*, with *F* set to zero for negative *F*^2^. The threshold expression of *F*^2^ > σ(*F*^2^) is used only for calculating *R*-factors(gt) *etc*. and is not relevant to the choice of reflections for refinement. *R*-factors based on *F*^2^ are statistically about twice as large as those based on *F*, and *R*- factors based on ALL data will be even larger.

### Fractional atomic coordinates and isotropic or equivalent isotropic displacement parameters (Å^2^)

**Table:**

	*x*	*y*	*z*	*U*_iso_*/*U*_eq_	
N1	−0.05463 (13)	0.58465 (7)	0.37647 (10)	0.0151 (2)	
N2	−0.25399 (14)	0.50164 (8)	0.16340 (12)	0.0204 (2)	
C1	0.11839 (16)	0.60745 (8)	0.50265 (12)	0.0151 (2)	
H1A	0.1274	0.6549	0.5629	0.018*	
C2	0.27885 (15)	0.56356 (8)	0.54419 (12)	0.0155 (2)	
C3	0.25605 (16)	0.49221 (8)	0.45170 (13)	0.0171 (2)	
H3A	0.3639	0.4586	0.4784	0.020*	
C4	0.08252 (16)	0.47011 (9)	0.32426 (13)	0.0177 (3)	
H4A	0.0711	0.4225	0.2631	0.021*	
C5	−0.07917 (16)	0.51858 (8)	0.28449 (12)	0.0159 (2)	
C6	0.47064 (16)	0.59045 (9)	0.68135 (13)	0.0196 (3)	
H6A	0.4574	0.6181	0.7487	0.029*	
H6B	0.5289	0.6378	0.6607	0.029*	
H6C	0.5502	0.5330	0.7238	0.029*	
O1	0.43909 (11)	0.88549 (6)	0.58761 (9)	0.0185 (2)	
O2	0.63811 (11)	0.83318 (6)	0.81350 (9)	0.0196 (2)	
N3	0.50078 (14)	0.75894 (8)	0.93299 (11)	0.0194 (2)	
C7	0.12951 (16)	0.86387 (9)	0.57403 (13)	0.0182 (2)	
H7A	0.1169	0.8984	0.5008	0.022*	
C8	−0.03024 (16)	0.84480 (9)	0.55754 (13)	0.0205 (3)	
H8A	−0.1507	0.8658	0.4747	0.025*	
C9	−0.01034 (16)	0.79390 (9)	0.66560 (13)	0.0194 (3)	
H9A	−0.1190	0.7788	0.6550	0.023*	
C10	0.16495 (16)	0.76523 (9)	0.78755 (13)	0.0171 (2)	
H10A	0.1751	0.7310	0.8599	0.021*	
C11	0.32976 (15)	0.78581 (8)	0.80692 (12)	0.0149 (2)	
C12	0.31006 (15)	0.83401 (8)	0.69541 (12)	0.0147 (2)	
C13	0.47372 (15)	0.85226 (8)	0.69945 (12)	0.0150 (2)	
H1	−0.280 (2)	0.4597 (12)	0.0936 (19)	0.032 (4)*	
H2	0.500 (2)	0.7164 (12)	0.9882 (18)	0.027 (4)*	
H3	0.605 (2)	0.7676 (13)	0.9397 (18)	0.034 (4)*	
H4	−0.165 (2)	0.6175 (13)	0.3487 (19)	0.038 (5)*	
H5	−0.354 (2)	0.5383 (12)	0.1417 (18)	0.031 (4)*	

### Atomic displacement parameters (Å^2^)

**Table:**

	*U*^11^	*U*^22^	*U*^33^	*U*^12^	*U*^13^	*U*^23^
N1	0.0141 (5)	0.0158 (5)	0.0159 (5)	0.0000 (4)	0.0099 (4)	−0.0003 (4)
N2	0.0149 (5)	0.0239 (5)	0.0183 (5)	−0.0009 (4)	0.0088 (4)	−0.0060 (4)
C1	0.0171 (5)	0.0140 (5)	0.0147 (5)	−0.0024 (4)	0.0104 (5)	−0.0017 (4)
C2	0.0150 (5)	0.0156 (5)	0.0153 (5)	−0.0013 (4)	0.0094 (5)	0.0012 (4)
C3	0.0158 (5)	0.0169 (5)	0.0201 (6)	0.0007 (4)	0.0123 (5)	0.0013 (5)
C4	0.0190 (6)	0.0167 (5)	0.0191 (6)	−0.0009 (4)	0.0130 (5)	−0.0028 (5)
C5	0.0163 (5)	0.0162 (5)	0.0156 (5)	−0.0018 (4)	0.0105 (5)	−0.0002 (4)
C6	0.0156 (5)	0.0200 (6)	0.0184 (6)	−0.0007 (4)	0.0087 (5)	−0.0018 (5)
O1	0.0187 (4)	0.0220 (4)	0.0151 (4)	−0.0006 (3)	0.0109 (4)	0.0014 (3)
O2	0.0132 (4)	0.0241 (5)	0.0180 (4)	−0.0006 (3)	0.0084 (4)	0.0046 (3)
N3	0.0150 (5)	0.0256 (5)	0.0152 (5)	−0.0005 (4)	0.0085 (4)	0.0043 (4)
C7	0.0179 (5)	0.0189 (6)	0.0146 (5)	0.0009 (4)	0.0090 (5)	0.0006 (5)
C8	0.0135 (5)	0.0250 (6)	0.0164 (6)	0.0020 (4)	0.0065 (5)	−0.0009 (5)
C9	0.0148 (5)	0.0219 (6)	0.0216 (6)	−0.0031 (4)	0.0117 (5)	−0.0053 (5)
C10	0.0185 (5)	0.0173 (6)	0.0176 (6)	−0.0032 (4)	0.0125 (5)	−0.0025 (4)
C11	0.0147 (5)	0.0138 (5)	0.0136 (5)	−0.0010 (4)	0.0079 (5)	−0.0027 (4)
C12	0.0139 (5)	0.0142 (5)	0.0144 (5)	−0.0017 (4)	0.0084 (5)	−0.0025 (4)
C13	0.0156 (5)	0.0124 (5)	0.0153 (5)	−0.0010 (4)	0.0091 (5)	−0.0013 (4)

### Geometric parameters (Å, º)

**Table:**

N1—C5	1.3498 (15)	O1—C13	1.2672 (14)
N1—C1	1.3660 (14)	O2—C13	1.2651 (14)
N1—H4	0.959 (18)	N3—C11	1.3717 (15)
N2—C5	1.3356 (15)	N3—H2	0.897 (17)
N2—H1	0.926 (18)	N3—H3	0.923 (18)
N2—H5	0.933 (17)	C7—C8	1.3821 (17)
C1—C2	1.3664 (16)	C7—C12	1.4064 (16)
C1—H1A	0.9500	C7—H7A	0.9500
C2—C3	1.4108 (17)	C8—C9	1.3966 (18)
C2—C6	1.5077 (16)	C8—H8A	0.9500
C3—C4	1.3716 (16)	C9—C10	1.3793 (16)
C3—H3A	0.9500	C9—H9A	0.9500
C4—C5	1.4125 (16)	C10—C11	1.4131 (16)
C4—H4A	0.9500	C10—H10A	0.9500
C6—H6A	0.9800	C11—C12	1.4153 (16)
C6—H6B	0.9800	C12—C13	1.5029 (15)
C6—H6C	0.9800		
			
C5—N1—C1	122.86 (10)	H6B—C6—H6C	109.5
C5—N1—H4	117.1 (11)	C11—N3—H2	117.4 (10)
C1—N1—H4	120.0 (11)	C11—N3—H3	117.0 (11)
C5—N2—H1	122.7 (10)	H2—N3—H3	121.8 (15)
C5—N2—H5	119.6 (10)	C8—C7—C12	122.28 (11)
H1—N2—H5	117.2 (15)	C8—C7—H7A	118.9
N1—C1—C2	121.54 (11)	C12—C7—H7A	118.9
N1—C1—H1A	119.2	C7—C8—C9	118.44 (11)
C2—C1—H1A	119.2	C7—C8—H8A	120.8
C1—C2—C3	116.59 (10)	C9—C8—H8A	120.8
C1—C2—C6	121.72 (11)	C10—C9—C8	120.90 (11)
C3—C2—C6	121.68 (10)	C10—C9—H9A	119.6
C4—C3—C2	121.81 (11)	C8—C9—H9A	119.6
C4—C3—H3A	119.1	C9—C10—C11	121.22 (11)
C2—C3—H3A	119.1	C9—C10—H10A	119.4
C3—C4—C5	119.61 (11)	C11—C10—H10A	119.4
C3—C4—H4A	120.2	N3—C11—C10	118.55 (11)
C5—C4—H4A	120.2	N3—C11—C12	123.23 (10)
N2—C5—N1	118.38 (10)	C10—C11—C12	118.22 (10)
N2—C5—C4	124.05 (11)	C7—C12—C11	118.86 (10)
N1—C5—C4	117.55 (10)	C7—C12—C13	118.47 (10)
C2—C6—H6A	109.5	C11—C12—C13	122.65 (10)
C2—C6—H6B	109.5	O2—C13—O1	123.53 (10)
H6A—C6—H6B	109.5	O2—C13—C12	118.48 (10)
C2—C6—H6C	109.5	O1—C13—C12	117.99 (10)
H6A—C6—H6C	109.5		
			
C5—N1—C1—C2	−0.49 (17)	C9—C10—C11—N3	177.35 (11)
N1—C1—C2—C3	−1.33 (17)	C9—C10—C11—C12	−2.10 (17)
N1—C1—C2—C6	178.25 (11)	C8—C7—C12—C11	−2.35 (18)
C1—C2—C3—C4	2.07 (17)	C8—C7—C12—C13	175.98 (11)
C6—C2—C3—C4	−177.52 (11)	N3—C11—C12—C7	−176.04 (11)
C2—C3—C4—C5	−1.02 (18)	C10—C11—C12—C7	3.38 (16)
C1—N1—C5—N2	−179.68 (11)	N3—C11—C12—C13	5.71 (18)
C1—N1—C5—C4	1.58 (16)	C10—C11—C12—C13	−174.87 (10)
C3—C4—C5—N2	−179.47 (11)	C7—C12—C13—O2	173.86 (10)
C3—C4—C5—N1	−0.81 (17)	C11—C12—C13—O2	−7.88 (17)
C12—C7—C8—C9	−0.13 (18)	C7—C12—C13—O1	−6.58 (16)
C7—C8—C9—C10	1.51 (18)	C11—C12—C13—O1	171.69 (10)
C8—C9—C10—C11	−0.38 (18)		

### Hydrogen-bond geometry (Å, º)

*Cg*1 is the centroid of the C7–C12 ring.

**Table:**

*D*—H···*A*	*D*—H	H···*A*	*D*···*A*	*D*—H···*A*
N3—H3···O2	0.92 (2)	1.97 (2)	2.6734 (18)	131.8 (15)
N2—H1···O1^i^	0.926 (18)	1.982 (18)	2.8561 (14)	157 (2)
N3—H2···O1^ii^	0.897 (17)	2.159 (18)	3.0445 (14)	168.7 (14)
N1—H4···O2^iii^	0.959 (18)	1.723 (18)	2.6776 (13)	172.7 (17)
N2—H5···O1^iii^	0.933 (17)	1.899 (18)	2.8305 (14)	176.8 (16)
C1—H1*A*···*Cg*1	0.95	2.58	3.5094 (13)	165

Symmetry codes: (i) −*x*, *y*−1/2, −*z*+1/2; (ii) *x*, −*y*+3/2, *z*+1/2; (iii) *x*−1, −*y*+3/2, *z*−1/2.
